# Assessing the Health-Care Risk: The Clinical-VaR, a Key Indicator for Sound Management

**DOI:** 10.3390/ijerph15040639

**Published:** 2018-03-30

**Authors:** Enrique Jiménez-Rodríguez, José Manuel Feria-Domínguez, Alonso Sebastián-Lacave

**Affiliations:** 1 Department of Financial Economics, Pablo de Olavide University, 41013 Seville, Spain; jmferdom@upo.es; 2 International Cooperation Andalusian Agency (AACID), Seville 41012, Spain; alonso.sebastian.ext@juntadeandalucia.es

**Keywords:** health-care, adverse event (AE), medical error (ME), clinical-value at risk, performance, quality improvement, key risk indicator (KRI), cost-effectiveness and efficiency measures

## Abstract

Clinical risk includes any undesirable situation or operational factor that may have negative consequences for patient safety or capable of causing an adverse event (AE). The AE, intentional or unintentionally, may be related to the human factor, that is, medical errors (MEs). Therefore, the importance of the health-care risk management is a current and relevant issue on the agenda of many public and private institutions. The objective of the management has been evolving from the identification of AE to the assessment of cost-effective and efficient measures that improve the quality control through monitoring. Consequently, the goal of this paper is to propose a Key Risk Indicator (KRI) that enhances the advancement of the health-care management system. Thus, the application of the Value at Risk (VaR) concept in combination to the Loss Distribution Approach (LDA) is proved to be a proactive tool, within the frame of balanced scorecard (BSC), in health organizations. For this purpose, the historical events recorded in the Algo-OpData^®^ database (Algorithmics Inc., Toronto, ON, Canada, IBM, Armonk, NY, USA) have been used. The analysis highlights the importance of risk in the financials outcomes of the sector. The results of paper show the usefulness of the Clinical-VaR to identify and monitor the risk and sustainability of the implemented controls.

## 1. Introduction

The concern about risk and its implications for patient safety has always been present in the agenda of health-care managers, although, traditionally it has dealt with a reactive vision rather than proactive management [1]. Based on the risk factor, it is split into clinical and non-clinical risk. The clinical risk includes any undesirable situation or factor related to the health-care environment that contributes to it, which can have negative consequences for the patient or be susceptible to generate what in medicine is known as adverse events (AEs) [2,3,4]. Therefore, the risk of the patient may be related—by commission or omission—to a failure to execute a plan or the wrong procedure to achieve a goal, that is, with a medical error (ME) [5]. In addition to generating risks to patient safety, these events can lead to large economic losses for the organization arising from compensation, leading to higher insurance premiums or loss of prestige and reputational risk [6]. On the other hand, the non-clinical risk is linked to the health organization’s management system, producing losses due to deliberate deviant behavior (e.g., internal fraud), or unintentional human errors (e.g., in data processing).

As Carey and Stefos [7] point out, the research on patient safety issues in the 1990s focused on widespread occurrence of AEs and their nature [8,9,10,11]. In this sense, although in 1980 the American Society for Healthcare Risk Management (ASHRM) was born, it was not until the publication of “*To Err is Human: Building a Safer Health System*” (hereinafter, the IOM report) [12] when risk management became a priority. This report found that 44,000 to 98,000 patients died each year in U.S. hospitals due to MEs, and on average the cost of non-fatal MEs stood between 17,000 and 19,000 million dollars annually. The IOM report [12] stimulated the development of several studies and discussion to understand and respond to AEs [13,14], to establish reporting systems for [15] or how quality could be adequately measured [16]. Following the release of the IOM report the rate of patient safety papers increased from 59 to 164 papers per 100,000 MEDLINE^®^ publications [17]; but this did not translate equally into better patient safety in practice [18], which still has a long way to go [19].

Therefore, the importance of health-care risk management is a current and relevant issue on the agenda of many institutions. Thus, the European Union (EU) estimates that between 8 and 12 percent of hospitalized patients suffer from AEs. In terms of costs, it amounts to an economic burden for the public health sector with direct costs of about €21 billion or 1.5% of health spending by EU member states. In the context of the financial crisis, the economic burden related with unsafe patient care received more attention [20]. In the last decade, some relevant research on economic evaluation of clinical risk have been published (see [7,21,22] for literature reviews).

The objective in the health-care sector has evolved from identifying AEs to determining cost-effective and efficient measures that improve control [23,24,25]. Consequently, the goal of the paper is to enhance the advancement of the hospital control system, through the development of a new Key Risk Indicator (KRI) within the frame of balanced scorecard (BSC). This planning and management control tool, proposed by Kaplan and Norton [26,27,28], enables to monitor the consequences arising from these actions by means of the integration of financial and non-financial measures. Moreover, its application is frequently limited to the use of performance measures (key performance indicator, KPI) without considering risk exposure measures (KRIs); which are presented in isolation. In this sense, this paper facilitates a comprehensive risk management framework for the healthcare organizations.

The BSC was designed according to four categories: financial performance, customer knowledge, internal business processes and learning and growth. Since then, the use and development of the BSC has been remarkable [29]. There is an interesting academic discussion on the theoretical foundations of the tool [30,31,32]. As Banchieri et al. [30] indicate the scientific production on BSC in the health-care sector has been profuse, while in practice, the implementation has been relatively slow, unlike other industries [33]. In the health sector, Naranjo-Gil [34] indicates it has not been easy to effectively implement the BSC due its complexity in relation to the immaturity of the management framework itself [35,36], but at the same time, many researchers have proposed it as a necessary tool in the hospital management strategy to improve quality and cost-effectiveness and have strived to perfect and adapt the tool for its practical application [37,38,39,40].

In this line of research, the contribution of the paper is to test the empirical application a proactive management tool to monitor the health-care risk. Specifically, both the Value at Risk (VaR) concept and Loss Distribution Approach (LDA) are proposed as a clinical a non-clinical risk metrics, indicating the maximum potential loss that could be incurred within a given period. Originally confined to actuarial discipline [41], the application of LDA to operational risk is explained by Embrechts and Puccetti [42]; in the next section, the methodological background is described. The empirical study has focused on the US health system. This study has been based on the losses due to operational events—clinical and non-clinical risk—recorded, from 1972 to 2009, in the database Algo-OpData^®^ provided by Algorithmics Inc. (IBM).

The results obtained show the financial impact of the health-care in the income. Therefore, analysis carried out on the performance of VaR highlight the utility of the estimating and monitoring such risk for the hospital management control system. The paper is structured as follows: Section 2 describes the methodology. Section 3 analyzes de data. The Section 4 and Section 5 summarizes the most important results and conclusions.

## 2. Methodology

Although risk is inherent to health activity and it is not possible to eradicate it, it can be managed, controlled and mitigated. In the academic field, there is abundant literature addressing the issue of risk management and patient safety [43,44]. Thus, it is assumed that the implementation of clinical risk management system improves the quality of care and the patient safety. Zineldin et al. [5] propose an integrated approach based on five quality dimensions (5Qs) for reducing medical errors and increasing patient safety [45].

In the strategy framework, the BSC allows identifying and monitoring the clinical risk ongoing and the sustainability of the risk control. The tool enables metrics to be linked together in a series of cause-and-effect relationships, affecting cost-effectiveness performance [46]. The classic BSC is based on the four main dimensions: financial performance, customer knowledge, internal processes and learning and growth. For each of them, the healthcare organization should define: (i) objectives; (ii) KPIs; (iii) measures; (iii) target; and (iv) initiatives. Consequently, the goodness of the BSC will be based on the usefulness of the previous indicators.

From an exhaustive literature review for the period 1975 to 2012, Azami-Aghdash et al. [25] identified 361 health-care indicators: 129 risk management indicators and 232 clinical effectiveness indicators; the highest number of indicators was related to patient safety.

In this paper, the BSC is enriched by introducing a new KRI based on the Value at Risk (VaR) concept. The adaptation of VaR to the hospital management system improves the supervision and control by linking the KPI to KRI, i.e., by integrating performance with risk management. Patient Wait Time, for example, is a frequently used KPI. In this regard, the objective of a healthcare organization should be to reduce the average amount of time that patient must wait between checking-in and being treated. But the checking-in process is exposed to potential errors—such as the data registration or find and attach medical history, for example—that could generate adverse event (AE) for the patient by providing an inadequate treatment. Consequently, the achievement of the targeted KPI must be linked to the success of the critical risk factor (KRI). Moreover, its implementation can improve the hospital’s management system.

VaR is accepted as a standard risk measure in the financial sector [47]. According to Embrechts et al. [48], there are four essential VaR applications: risk management, financial control, financial reporting and estimating regulatory capital in banking. Although its usefulness in the health sector can be large, e.g., Dehlendorff et al. [49] apply the Conditional Value at Risk (CVaR) as an indicator of hospital waiting time.

Mathematically, for a given confidence level α∈(0, 1), *VaR* is defined as follows:

(1) $$VaR_{\propto }=inf\{l\in R:P\left(L>l\right)\leq 1-\alpha \}=inf\left\{l\in R:F_{L}\left(l\right)\geq \alpha \right\}$$

where the probability *P* that the loss *L* exceeds the value *l* will not be higher than (1 − *α*).

Although this approach comes from the financial risk management field, it can be transposed to any other risk-exposed industries, such as healthcare. It indicates the maximum expected loss that a hospital or clinical management unit could incur due to the risks associated to its processes, during a period and within a confidence interval established; in this paper, it is named as Clinical-VaR or Non-Clinical-VaR. Thus, for financial control and management purposes, parameters *α* for VaR as 1%, 5% or 10% and times horizon quarterly, semesters or annual could be suitable combinations.

**Figure 1:**
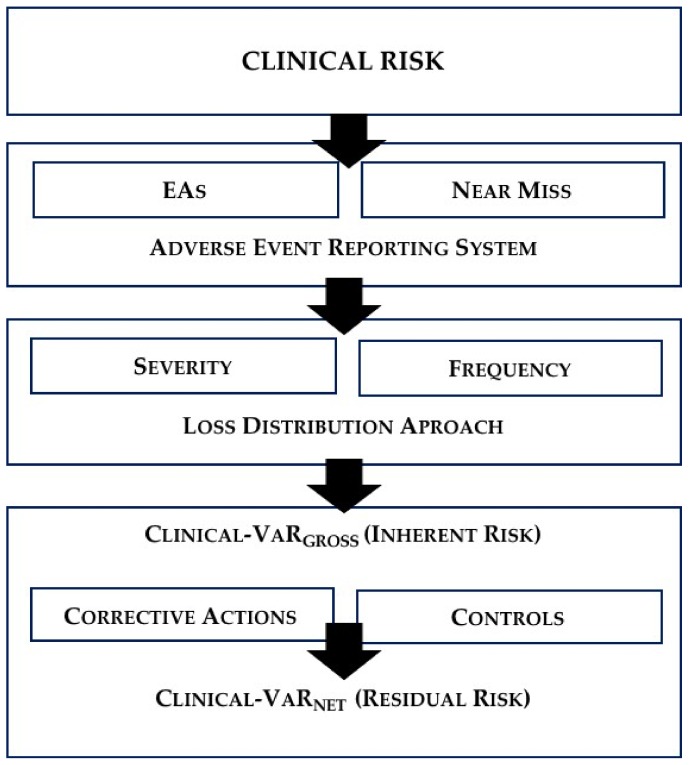
Clinical-VaR process.

In any case, for the health-care manager, Clinical-VaR shouldn’t be a simple figure, it must be a management system. As Jorion [50] points out, the main goodness of a VaR-based risk management is the continuous improvement of processes. The system is to run periodically estimating the Clinical-VaR, that is, exposure to inherent risk. This risk metric forces hospital managers to set up corrective actions or controls, improving the management system. These improvements can be transferred to the BSC through risk scores, thus reflecting the residual risk or the Clinical-VaR_Net_ (see Figure 1).

### 2.1. Loss Distribution Approach

Since VaR is a percentile applied to the distribution function of a certain variable, in the case of AEs it is linked to the Loss Distribution Approach (LDA). Developed for Bühlmann [41], this statistical technique aims to obtain a probability distribution of aggregate losses. The model is based on historical events from which the function of both severity and frequency are estimated. Consequently, the implementation of Clinical-VaR as a management tool is linked to the development of an Adverse Event Reporting System (AERS) that allows building a database.

### 2.2. Frequency Model

The random variable N represents the number of events occurred in a determined time known as risk horizon τ. The loss frequency distribution P_t_ is a discrete variable defined by:

(2) $$P_{t}\left(n\right)=\overset{n}{\underset{k=0}{\sum }}p\left(k\right)$$

where p represents the probability function of events (k).

A priori, any nonnegative discrete probability distribution would be a potential candidate to fit the frequency [51]. Fountnouvelle et al. [52] suggest the Poisson distribution (Po) as the best starting point to modelling recurring and random events. The Poisson is characterized by a single parameter, λ, representing the mean and the variance of the distribution. In this study, the Negative-Binomial (NB) and Geometric (Ge) distributions are also showed; commonly used in the modelling of the frequency of operational risk [53]. These functions are described in detail below:

(3) $$N\sim \mathrm{Po}\left(\lambda \right)\to P\left(N=k\right)=\frac{\lambda^{k}e^{-\lambda }}{k!}\;\;\lambda >0,\;k=0,1,2,\ldots$$

(4) $$N\sim \mathrm{NB}\left(r,p\right)\to P\left(N=k\right)=\left(\begin{array}{c} k+r-1\\ k \end{array}\right)p^{k}{\left(1-p\right)}^{r}\;\;r>0,\;p\in \left(0,1\right)$$

(5) $$N\sim \mathrm{Ge}\left(p\right)\to P\left(N=k\right)={\left(1-p\right)}^{k-1}p\;r>0,\;p\in \left(0,1\right)$$

### 2.3. Severity Model

Severity refers to the economic impact caused by an AE (clinical risk) or other operatives and management events (non-clinical risk). The severity is defined as: “a continuous random variable, (*X_k_*)*k* ∈ *N*, that takes positive values, which are independent of each other, and identically distributed (iid)” [54]. Panjer [51] provides a compendium of functions that could be used. In practice, the sample of events shows both high asymmetry and kurtosis. In order to determine which probabilistic function best fit the data, Dutta and Perry [55] suggest:

1. Goodness of fit (GOF): Statistically, which is the degree of adjustment of adverse event data related to the distribution?
2. Realistic: The estimated Clinical-VaR through the model; is it a realistic vision? Does it make economic sense?
3. Well specified: are the characteristics of the theoretical distribution and the ones of the empirical sample of loss events consistent?
4. Flexible: Can the model be extrapolated to a wide range of samples of losses due to clinical risk?
5. Simple: Is the implementation of the model workable from the hospital management system?

Moscadelli [56] considers studying the distributions according to theirs kurtosis: he proposes the Weibull function for soft tail; the Lognormal or Gumbel in medium tail; and for the ones of heavy tails the Pareto’s function. On the other hand, Fountnouvelle et al. [52] agree on the reasoning, so for heavy-tail distributions suggest the Pareto, Pareto-generalized, the Burr, the Log-Logistic and the Log-Gamma functions. In line with the above-mentioned studies, it seemed appropriate to use a range of functions with features different in terms of their tail. Thus, the selected candidates are the following: Burr (Bu), Inverse-Gaussian (IG), Log-Logistic (LL), Log-Normal (LN), Normal (N), Pareto (Pa) and (We) Weibull. The Normal function has been included to discard the Gaussian path of operational losses in the health-care organizations.

(6) $$X\sim \mathrm{Bu}\left(a,b,c,d\right)\to f\left(x\right)=\frac{\mathrm{cd}}{{\mathrm{bz}}^{c+1}{\left(1+z^{-c}\right)}^{d-1}},\;b>0,\;c>0,\;d>0,\;z=\left(\frac{x-a}{b}\right),\;x>a$$

(7) X~IG(μ,θ)→f(x)=(θ2πx3)12exp(−θ2x(x−μμ)2)  μ>0,θ>0

(8) $$X\sim \mathrm{LL}\left(\alpha ,\beta \right)\to f\left(x\right)=\frac{\alpha }{\beta }{\left(\frac{x}{\beta }\right)}^{\alpha -1}{\left(1+{\left(\frac{x}{\beta }\right)}^{\alpha }\right)}^{-2}\;\;\alpha >0,\beta >0$$

(9) $$X\sim \mathrm{LN}\left(\mu ,\sigma \right)\to f\left(x\right)=\frac{1}{x\sigma \sqrt{2\pi }}\exp\left[-\frac{1}{2}{\left(\frac{\ln x-\mu }{\sigma }\right)}^{2}\right]\;\mu \in R,\;\sigma >0$$

(10) $$X\sim N\left(\mu ,\sigma \right)\to f\left(x\right)=\frac{1}{\sigma \sqrt{2\pi }}\exp\left[-\frac{1}{2}{\left(\frac{x-\mu }{\sigma }\right)}^{2}\right]\;\;\mu \in R,\;\sigma >0$$

(11) $$X\sim \mathrm{Pa}\left(\alpha ,\beta \right)\to f\left(x\right)=\frac{{\alpha \beta }^{\alpha }}{x^{\alpha +1}}\;\;\;\;\alpha >0,\beta >0$$

(12) $$X\sim \mathrm{We}\left(\alpha ,\beta \right)\to f\left(x\right)=\frac{\alpha }{\beta }{\left(\frac{x-\gamma }{\beta }\right)}^{\alpha -1}\exp{\left(-{\left(\frac{x-\gamma }{\beta }\right)}^{\alpha }\right)}^{}\;\alpha >0,\beta >0,\gamma \equiv 0$$

### 2.4. Goodness of Fit

Since the Clinical-VaR is a statistical estimate, the success of its application will depend on determining the most suitable probability distribution to the existing data. Thus, once the families of distributions have been proposed, the consistency of these with the empirical distribution function (EDF) must be tested. The contrast of Kolmogorov-Smirnov (KS) is recommended in the study of continuous variables. The KS statistic is denoted by “*D*” and represents the maximum distance, in absolute terms, between the theoretical, $\hat{F_{n}}\left(x\right)$, and empirical, $F_{n}\left(x\right)$, distribution function:

(13) $$D=\max\;\left\{D^{+},D^{-}\right\}$$

where $D^{+}=\sqrt{n}\sup_{x}\left\{F_{n}\left(x\right)-\hat{F_{n}}\left(x\right)\right\}$ and $D^{-}=\sqrt{n}\sup_{x}\left\{\hat{F_{n}}\left(x\right)-F_{n}\left(x\right)\right\}$. To make comparable samples of different sizes, the supremum class statistics are multiplied by $\sqrt{n}$ and the quadratic class by *n* [57]. The resulting statistic *D* has been compared with the appropriate critical value for a stated level of significance (α) and is denoted by *d_α_*. In expression (14) are represented the null, $H_{0}$, and alternative, $H_{A}$, hypothesis:

(14) $$\begin{array}{c} H_{0}:F_{n}\left(x\right)\in \hat{F_{n}}\left(x\right)\\ H_{A}:F_{n}\left(x\right)\notin \hat{F_{n}}\left(x\right) \end{array}$$

so if *D ≤ d_α_*, $H_{0}$ is accepted for a level of significance α, in other words, if *p*-value ≥ *α* → accept $H_{0}$. In addition, to validate the choice of distribution and therefore to reduce the risk of the model, it has been fit to be supported by graphical tools such as the Probability-Probability (PP) Plots.

### 2.5. Estimation of the Aggregated Loss Distribution

Having described the distributions of severity and frequency, the aggregated losses distribution (ALD) is obtained by compounding of both. If the aggregate losses are denoted as a sum, *S*, associated to a set of *N* observed individual losses, ($X_{1}+X_{2}+\cdots +X_{N}$), being $S=X_{1}+X_{2}+\ldots +X_{N},N=0,1,2\ldots ,$ where, *X_i_* is the severity and N the number of events in the time interval [0, t], i.e., the event count. Assuming the severity and the frequency processes are independent [58], the aggregated loss distribution is defined as:

(15) $$S=\overset{N\left(t\right)}{\underset{i=0}{\sum }}X_{i},\;t\geq 0$$

The distribution function of *S* is denoted as F. The cumulative distribution function of the loss aggregated is represented as $G_{t}\left(x\right)$:

(16) $$G_{t}\left(x\right)=f\left(x\right)=\{\begin{array}{ll} \sum_{n=0}^{\infty }p_{t}\left(n\right)F_{t}^{n^{*}}\left(x\right) & x>0\\ p_{t}\left(0\right) & x=0 \end{array}$$

The asterisk denotes the convolution in function F, where $F^{n^{*}}$ *n*-times is the convolution of F with itself, i.e.:

(17) $$\begin{array}{c} F^{1^{*}}=F\\ F^{n^{*}}=F^{{\left(n-1\right)}^{*}}*F \end{array}$$

Convolution is a mathematical process that converts the frequency and severity distributions on third distribution (ALD) by superimposing both [59]. For the estimation of the $G_{t}\left(x\right)$ a Monte-Carlo simulation has been applied:

1. A random sample of the frequency distribution is generated, i.e., *n* loss events per year are simulated.
2. *N* random samples of the distribution of severity are taken: *L*_1_ + *L*_2_ + *L*_3_+ … + *L_n_*
3. The computation of the n simulated losses represents the total loss: *X* = *L*_1_ + *L*_2_ + *L*_3_ + … + *L_n_*
4. Return to step 1, and it is repeated m times: *X*_1_, *X*_2_, *X*_3_, …, *X_m_*

Therefore, *m* represents the number of generated simulations. In this study, for each of the convolutions made it has been made a million simulations, obtaining relative errors well below 1%.

### 2.6. Clinical-Value at Risk

For the Clinical-VaR and Non-Clinical-VaR calculation only apply the selected percentile to ALD. In this case, VaR is an internal variable management in the health-care, consequently, the risk managers may select the percentile discretionally based on their management criterion. In the study, there have been selected the 90, 95 and 99 percent to analyze their impact. Expression (18) represents the synthetic Clinical-VaR formula:

(18) $$\mathrm{Clinical}­\mathrm{VaR}=G^{-1}\left(\alpha \right)$$

## 3. Data and Sample

### 3.1. Description and Sample Selection

Coile [60] indicates that between 2.9 and 3.7 percent of hospitalized patients suffer an AE as a result of some medical malpractice event. In this line, there are 10 percent of patients who have suffered some AE due to the performance of the health workers [61,62], where 90 percent of the cases were avoidable errors [63,64]. On the other hand, events that by chance, prevention or mitigation do not generate adverse effects on the patient are categorized as near misses [62].

There are operational risks in the health-care process that do not generate AEs for the patient safety, although these events do generate large losses to the organization, that is, they represent a non-clinical risk. Thus, Rosenbaum et al. [65] estimated that 80% of fraud events are carried out by medical providers, by patients 10% and the rest by insurance companies and their employees. In economic terms, the costs incurred by fraud, abuse and excessive payments for services can reach 10 percent of the total expenditure on health in the U.S. [66]. Byrd et al. [67] highlighted the difficulty of establishing a certain amount of the cost of fraud due to the underreporting of this type of events. In any case, they range from $100 billion to $600 billion in the health sector; consequently, fraud is a major risk in the management of a health organization.

Due to the immaturity of the AERS, most hospitals do not have a sufficiently extensive AE database (long-term records) to support robust models. The absence of data does not mean absence of risk, so in order to address the lack of hospital’s internal data Scenario Analysis is commonly applied. Designing scenarios is based on external databases, expert opinions in the field or risk indicators.

Thus, the external database Algo-OpData^®^ provided by Algorithmics Inc. (IBM) is used. Based on regulatory reports, court and legal documents, consulting reports and business publications, this database collects operational events of the main sectors of the economy with a minimum threshold of one million dollars. In scientific literature, some robust papers rely on the Algo-OpData^®^ [68,69].

**Figure 2:**
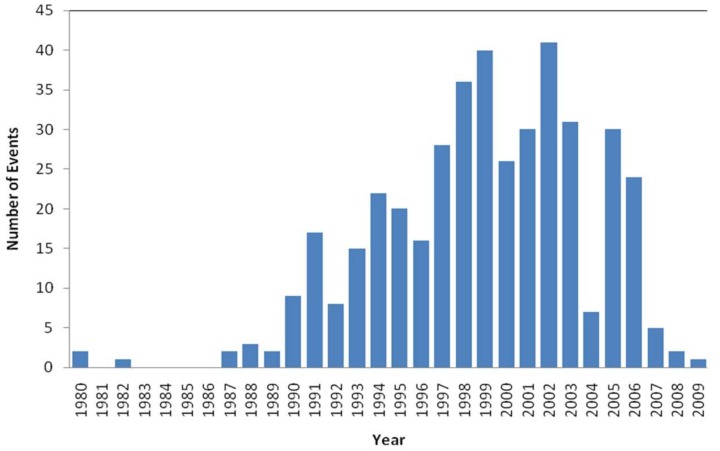
Histogram of the annual frequency.

**Figure 3:**
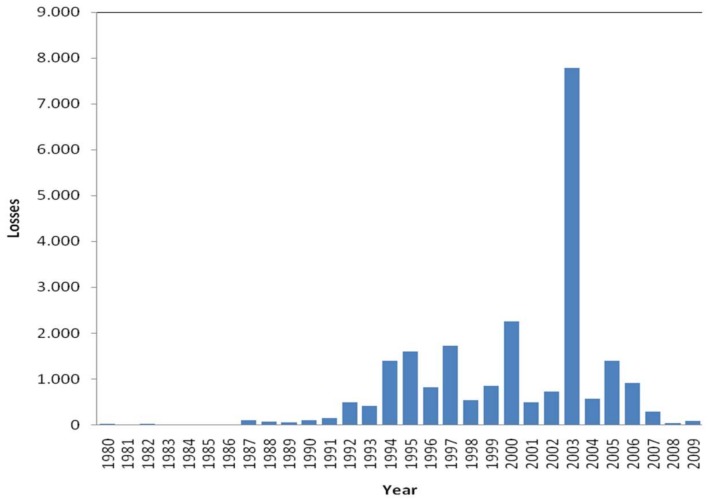
Annual losses in dollars.

The sample consists of those events related to the health sector and located in the United States. Figure 2 and Figure 3 show the number of events and the sum of the losses distributed per year. On the other hand, although the database collects events from 1980 to 2009, in order not to distort the real frequency, the sample has been limited since the year 1987; the number of events logged before 1987 are slightly significant (see Figure 2). Therefore, although the database includes 12,314 events, a first selection of 418 events is made, whose total losses reached $23 billion. In Figure 3, the breakdown of this amount for each year of the sample is appreciated; in 2008, the number of losses reached 8 billion dollars. Also, an operational loss may take months or years to mature. Thus, there is likely to be events that are developing but have not yet discovered. For example, between 1992 and 2002, at the Tenet’s Redding Medical Center, cardiac catheterization and unnecessary bypass surgeries were performed on 796 patients. Years later, after a collective legal action, Tenet Healthcare Corporation paid 395 million dollars to the affected patients. In this respect, Chernobai et al. [70] indicate that the average operational risk event duration is about 20 months, it doesn’t last more than 48 months 75 percent of the time. If data in 2006 are censured, at least 50% of the registered events are contemplated. Finally, the selected events have been restricted to 407 and structured into the subsets: clinical and non-clinical risk.

In 2003, a striking peak due to an extraordinary event can be observed: Medicare fraud comes into light. Medicare is a national social insurance program administered by the US federal government, which provides medical care to all people over 65 years of age or younger who are considered disabled due to serious health problems. Thus, certain medical providers—among them the Tenet Health Corporation group—had been performing a massive fraud for years, based on billing charges to Medicare for medications and treatments that were not necessary, or even were not carried out. The fraud reached the amount of 6 billion dollars.

### 3.2. Exploratory Data Analysis

As Tukey [71] argues before testing what probability distribution is best suited to the shape of the sample, it is suitable to carry out a rigorous Exploratory Data Analysis (EDA). Dutta and Perry [55] and Moscadelli [56], as a preliminary step in its study on the measurement of operational risk, perform the EDA on the data sample. Previously, authors such as Badrinath and Charrerjee [72] had carried out such an analysis in studies of share price data. In this study must focus on contrasting the absence of normality and on evaluating the skewness and kurtosis of the empirical distribution; a priori, characterized by heavy tails [56]. To achieve this point, the guidelines marked by Tukey [71] and Hoaglin et al. [73] have been followed. The study of the descriptive statistics of the frequency and severity is shown in Table 1 and Table 2, respectively.

As observed in Table 1, both the clinical and the non-clinical risk samples show over-dispersion, that is, the variance is greater than the average of annual events. Regarding the severity (Table 2), if the measures of central tendency are observed, in both cases the mean is greater than the median, accentuating the asymmetry and discarding the hypothesis of normality. This particular characterization will condition the goodness of fit.

## 4. Results

### 4.1. Modelling

Following the methodological process described in Section 2, first, the models that best fit the empirical data of frequency and severity are determined. The function parameters are estimated by maximum likelihood. Alpha is chosen by convenience; depending on the degree of conservatism of the analysis three levels are established, that is: 0.1, 0.05 and 0.01; giving raise to three confidence intervals different 90%, 95% and 99%, respectively (see Table 3 and Table 4).

Bounded critical values, Table 5 and Table 6 show the Kolmogorov-Smirnov test result for frequency and severity, respectively.

Table 5 shows that Ge function as the best performer with a significance level above 10 percent in both cases (0.4913 and 0.1411). The geometric distribution is a unifactorial function that implies the independence of the events among themselves. However, the Po function has the lowest statistical value. The frequency of operational events in the health-care process present over-dispersion (see Table 1), hence the Po function assuming equidispersion give a lower significance of 0.0505 and 0.0309, respectively. The NB function is sensitive to overdispersion, so a significant fit for clinical risk (0.3578) is observed, although it is low for non-clinical (0.0451). The results of the Kolmogorov-Smirnov test for severity are detailed in Table 6. The loss distributions of the two samples show common elements, namely positive asymmetry and leptokurtosis, although the intensity of these statistical moments is different in each (see Table 2). This is evident in the results the GOF. The clinical risk setting presents a high degree of significance; the Burr function obtains a *p*-value of 0.7311. However, in non-clinical risk the Bu function does not reach a minimum of acceptable significance, in this case the log-normal is the model that best fit with a statistical value of 0.0809 and a *p*-value of 0.0274. In Table 6 we can notice that as the normality assumption is discarded, the probability that K-S statistical is less than its critical value of zero or a value closer to zero.

**Figure 4:**
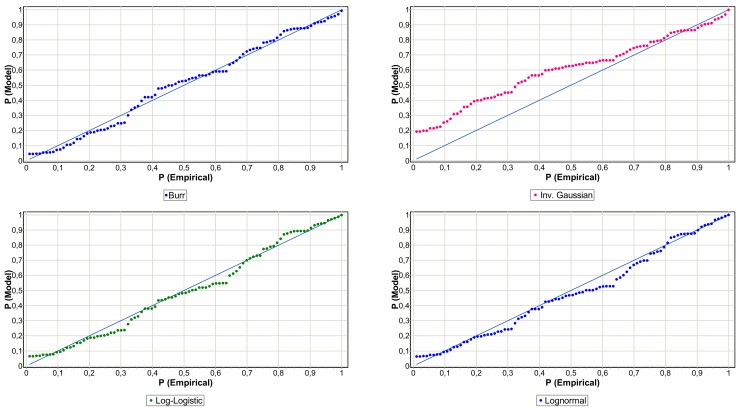

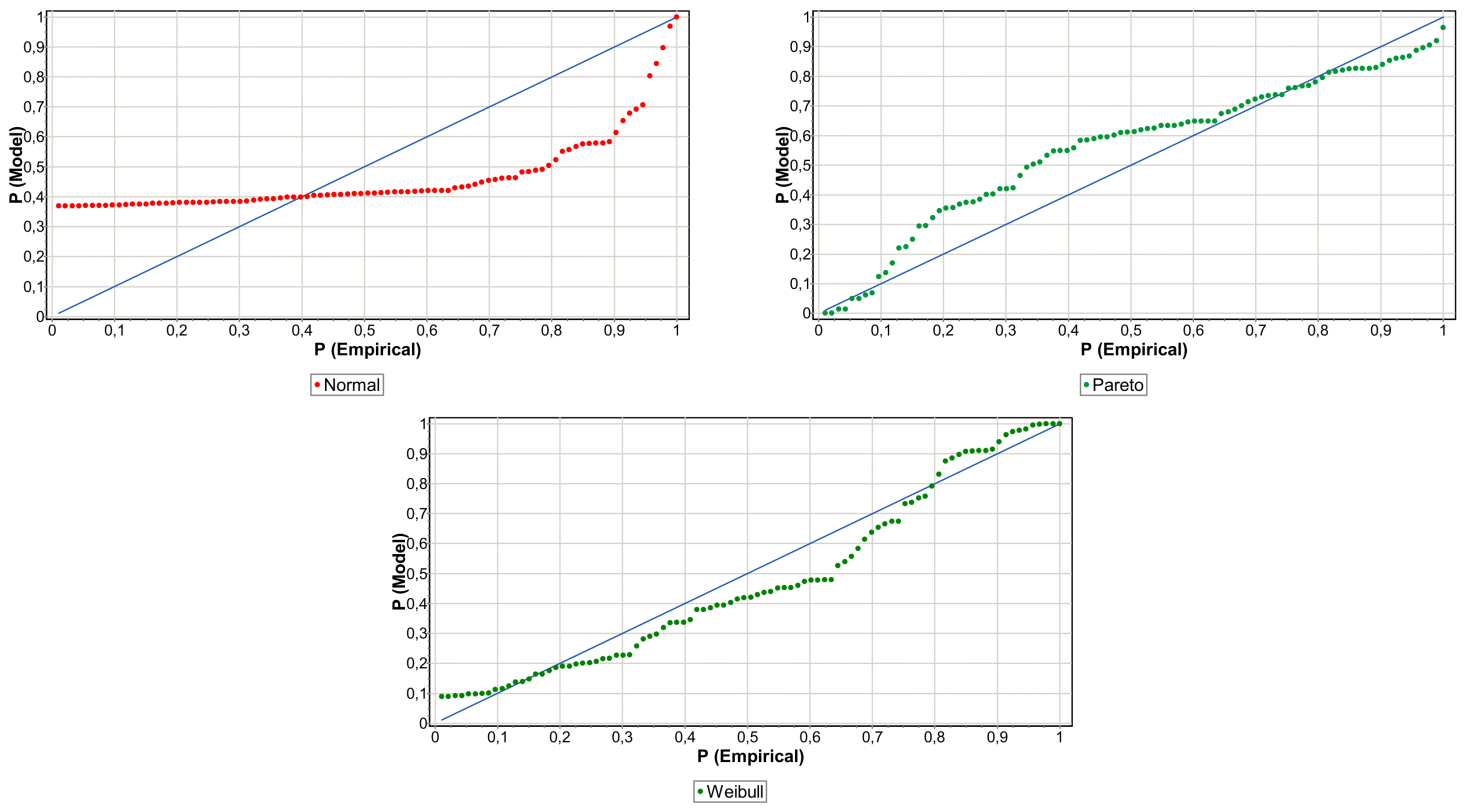
Clinical-risk P-P plots of severity distributions.

**Figure 5:**
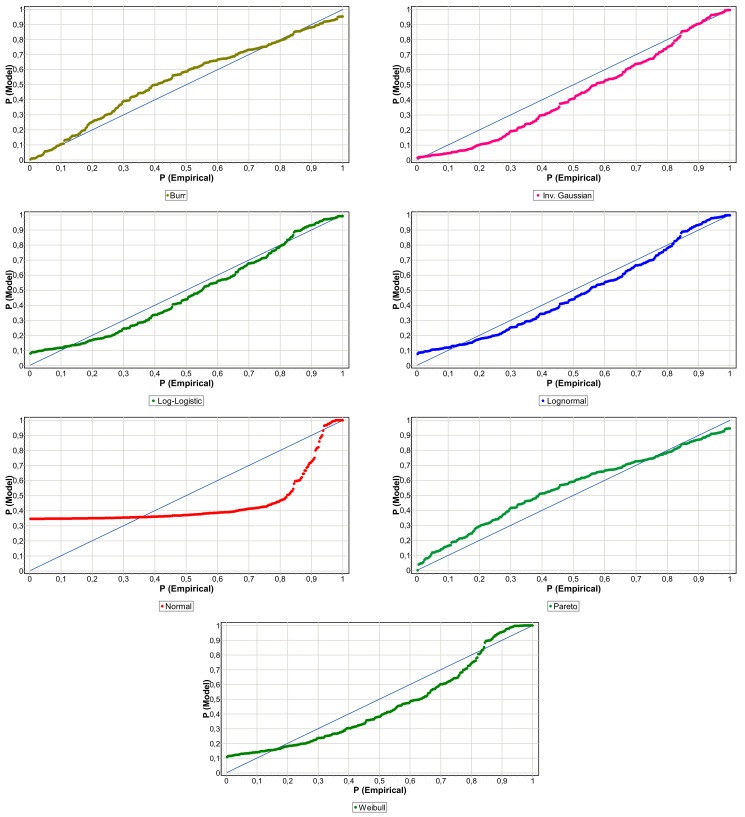
Non-clinical risk P-P plots of severity distributions.

In Figure 4 and Figure 5, to add arguments to the choice the analysis of probability-probability (PP) plot for the two samples is performed, comparing the empirical with the theoretical distribution. The P-P plots further justify the choice of the Burr function for clinical-risk (see Figure 4) and Log-Normal function for non-clinical risk (see Figure 5), since they are closer to the empirical distribution. The graphic analysis also allows us to corroborate that the Log-Normal (*p*-value equal to 0.2237) and Log-Logistic (*p*-value equal to 0.4674) would also be valid to model the severity of clinical risk events; Figure 4 shows that the points are located closely along the straight line. Regarding Figure 5, in the non-clinical risk, only Log-Normal and Log-Logistic reach a *p*-value higher than 0.5% of significance and with an acceptable graphic fit. The low GOF of the non-clinical risk is explained by the heterogeneity of the sample, you observe the high asymmetry and fat tail; skewness and kurtosis are respectively equal to 15.91 and 275.15.

### 4.2. Clinical-VaR and Non-Clinical-VaR

**Figure 6:**
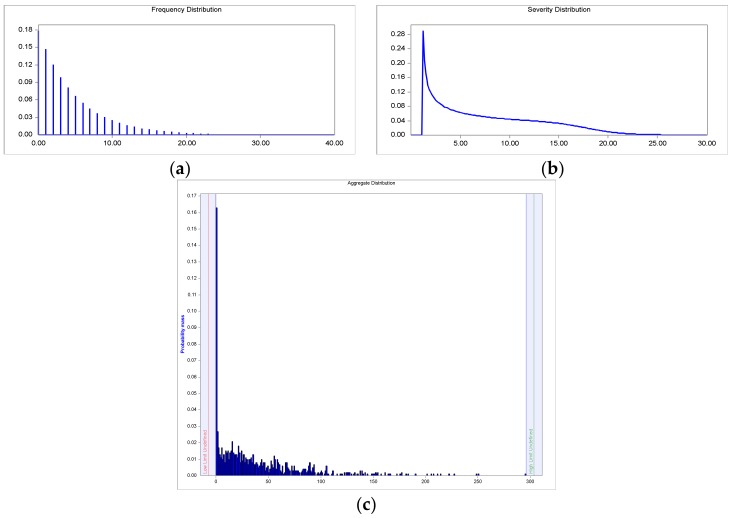
Clinical-VaR: (**a**) Frequency histogram of the geometric distribution; (**b**) Probability density function (PDF) of the Burr distribution; (**c**) Loss distribution approach (LDA).

**Figure 7:**
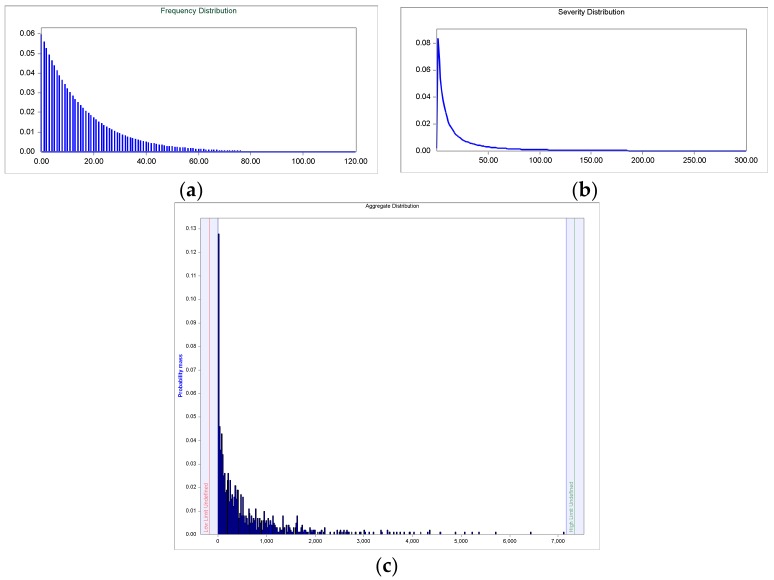
Non Clinical-VaR: (**a**) Frequency histogram of the geometric distribution; (**b**) Probability density function (PDF) of the Log-Normal distribution; (**c**) Loss distribution approach (LDA).

Therefore, for the determination of the aggregate distribution two models are built: Bu & Ge (clinical risk) and LN & Ge (non-clincal risk), whose convolution processes are illustrated in Figure 6 and Figure 7, respectively. As seen in the frequency graphs (Figure 6a and Figure 7a), where in both cases, the geometric function adjusts the over-dispersion of the events; the variance is higher than the mean.

Regarding severity, in Figure 6b and Figure 7b, the theoretical model of the Burr and the log-normal function illustrate the asymmetry and leptokurtosis of the empirical distributions. In any case, ALD is sensitive to the greater fat tail appreciated in the non-clinical risk, see Figure 7c, versus the clinical risk, see Figure 6c; this aspect determines the VaR calculations. The results of the VaR calculation are shown in Table 7. In general, much higher figures are observed in the non-clinical versus the clinical risk; this is due, especially, to the fat tail of its events sample. The valuations are made for the one year time horizon. Thus, the mean represents the expected economic costs—in billions of dollars—for each type of risk; 38.64 for clinical risk and 575.13 for non-clinical risk. The VaR has been estimated for three confidence levels, that is: 10, 5 and 1 percent. In light of the above data, the sensitivity analysis on the VaR shows the strong impact that produces the alpha parameter. The Clinical-VaR takes the following values: 92.67; 127.05; and 190.85. In the non-clinical risk, values much more stressed for the VaR were obtained: 1533.90; 2116.90; and 3278.60. In this sense, managers must be cautious when setting the level of confidence; a high degree of conservatism could give unrealistic figures in a leptokurtic environment.

### 4.3. Discussion

Risk is intrinsic to the hospital processes. When the clinical risk materializes it can generate an AE, that is, negative effects for patient safety. The analysis of the events recorded in the Algo-OpData^®^ database highpoints the importance of these incidents for the patient’s health, but also their economic effects. In addition to AEs, those failures that do not imply a direct risk for the patient although do generate expensive losses for the health system has been identified as a non-clinical risk [66]. In the 20 years of the selected sample, the losses due to non-clinical risk in the US health system exceeded 20 billion dollars and for clinical risk 1.5 billion dollars. Note that, this study only takes into account those events that exceeded one million dollars. Either way, a special consideration is given to fraud events that involve deliberate deviant behavior by medical providers, patients, insurance companies or their employees [65]. Consequently, concern for risk is a key element in hospital management and should be integrated into the health-care management system.

From the review of the literature, there is a turning point in the treatment of clinical risk after the publication of IOM report [12]. This evolution is evident in the research topics that evolve from focusing on the identification of the nature of the AE, to proposing improvements in management and quality health-care systems [8,9,10,11,12,13,14,15,16].

As indicated in previous paragraphs, over the last few years, different KPIs have been implemented, this has meant an improvement in the quality of the hospital service. In this context, a key risk indicator has been tested, that is, the Clinical-VaR. The VaR is a metrics contrasted by the academics and practitioners and it is consolidated as a rigorous financial management tool [47,48,49,50,54]. Its adaptation to the health sector allows us to control, for example, waiting times, as proposed by Dehlendorff et al. [49]. The contribution of this paper is based on the application of the VaR to project and control the AEs, as well as its economic consequences. Technically, the Clinical-VaR is a figure resulted of applying a certain percentile to the aggregate distribution of events, convolution of severity and frequency functions. As the results show (Table 7), the impact of the determined percentile is remarkable. Since alpha establishes the confidence interval of the estimation, the determination of a VaR_90_ or VaR_99_ is discretionary and conditioned to the degree of conservatism that the manager wants to apply.

The application of the VaR within the framework of the balanced scorecard is proposed. So, in the second stage, the objective for the management is to evaluate the quality of the controls and the corrective actions applied, based on the comparison of the VaR_GROSS_ figure, inherent risk, and the VaR_NET_, residual risk.

Note that the main handicap for its effective implementation is the lack of depth of the adverse events databases. Despite the fact that many efforts are being made in the development of AERS that allow the building of databases, there are an inhibitors that cause underreporting [15]. So, our future research lines shall focus on improving the AERS.

## 5. Conclusions

In this study, the application of the LDA model and the VaR metric to assess risk in the health-care process was empirically tested. Thus, the clinical risk was isolated from the non-clinical, establishing a new KRI called Clinical-VaR and Non-Clinical-VaR, in the second case. Clinical-VaR indicator allows projecting the maximum number of expected AEs and their financial costs, for a given time horizon and confidence interval. The implementation of this management tool is conditioned to the availability of historical data of events with sufficient depth to obtain significant results. Only the recognition of the causes of the AEs allows adopting effective strategies to reduce them. In this line, the inclusion of Clinical-VaR in the BSC facilitates the identification and control risk ongoing and quality controls with the comparison of inherent and residual risk. The Clinical-VaR not only can measure the potential losses from process failures but also it helps managers to monitor the potential deviations over the targeted levels. Consequently, the usefulness of the proposed indicator is clear and in line with the strategy of improving the quality of health-care and the patient safety.

## Figures and Tables

**Table 1:** Descriptive statistics of frequency.

	Mean	Median	Variance	Skewness	Kurtosis	CofV
Clinical Risk	4.6	4.00	25.62	1.96	8.04	1.10
Non-Clinical Risk	15.75	18.00	93.25	−0.04	1.87	0.61

**Table 2:** Descriptive statistics of severity.

	Mean	Median	Variance	Skewness	Kurtosis	CofV
Clinical Risk	17.11	6.35	228.10	7.93	72.87	2.79
Non-Clinical Risk	65.64	7.95	1610.10	15.91	275.15	6.11

**Table 3:** Critical value test of frequency distribution.

	*d*_0.90_	*d*_0.95_	*d*_0.99_
Clinical & Non-Clinical Risk	0.2647	0.2941	0.3524

**Table 4:** Critical value test of severity of distribution.

	*d*_0.90_	*d*_0.95_	*d*_0.99_
Clinical Risk	0.1251	0.1389	0.1667
Non-Clinical Risk	0.0681	0.0756	0.0906

**Table 5:** Kolmogorov-Smirnov test of frequency distribution.

	Clinical Risk KS Statistic	Non-Clinical Risk KS Statistic
Geometric	0.1786 (0.4913)	0.2488 (0.1411)
Neg-Binomial	0.1991 (0.3578)	0.2982 (0.0451)
Poisson	0.2937 (0.0505)	0.3128 (0.0309)

**Table 6:** Kolmogorov-Smirnov test of severity distribution.

	Clinical Risk KS Statistic	Non-Clinical Risk KS Statistic
Burr	0.0696 (0.7311)	0.1069 (<0.005)
Inv-Gaussian	0.2098 (<0.005)	0.1288 (<0.005)
Log-Logistic	0.0863 (0.4674)	0.0837 (0.0205)
Log-Normal	0.1067 (0.2237)	0.0809 (0.0274)
Normal	0.36927 (<0.005)	0.3444 (0)
Pareto	0.18252 (<0.005)	0.11966 (<0.005)
Weibull	0.1555 (0.0198)	0.1349 (<0.005)

**Table 7:** Clinical-VaR and Non-Clinical-VaR.

	Severity	Frequency	Mean	VaR_0.90_	VaR_0.95_	VaR_0.99_
Clinical VaR	$Bu\left(\begin{array}{c} \begin{array}{c} a=1.16\\ b=17.34\\ c=9.77 \end{array}\\ d=0.06 \end{array}\right)$	Ge(p=0.18)	38.64	92.67	127.05	190.85
Non-Clinical VaR	$LN\left(\begin{array}{c} \mu =37.22\\ \sigma =129.52 \end{array}\right)$	Ge(p=0.06)	575.13	1533.90	2116.90	3278.60
